# Prevalence of Antibiotic Resistance Over Time in a Third-Level University Hospital

**DOI:** 10.1089/mdr.2021.0109

**Published:** 2022-04-18

**Authors:** Vincenzo Scaglione, Mariaconcetta Reale, Chiara Davoli, Maria Mazzitelli, Francesca Serapide, Rosaria Lionello, Valentina La Gamba, Paolo Fusco, Andrea Bruni, Daniela Procopio, Eugenio Garofalo, Federico Longhini, Nadia Marascio, Cinzia Peronace, Aida Giancotti, Luigia Gallo, Giovanni Matera, Maria Carla Liberto, Bruno Mario Cesana, Chiara Costa, Enrico Maria Trecarichi, Angela Quirino, Carlo Torti

**Affiliations:** ^1^ Unit of Infectious and Tropical Diseases, Department of Medical and Surgical Sciences, “Magna Graecia” University of Catanzaro, Catanzaro, Italy.; ^2^ Unit of Clinical Microbiology, Department of Health Sciences, “Magna Graecia” University of Catanzaro, Catanzaro, Italy.; ^3^ Unit of Intensive Care, Department of Medical and Surgical Sciences, “Magna Graecia” University of Catanzaro, Catanzaro, Italy.; ^4^ Unit of Medical Statistics, Biometrics and Bioinformatics “Giulio A. Maccacaro”, Department of Clinical Sciences and Community Health, Faculty of Medicine and Surgery, University of Milan, Milan, Italy.; ^5^ Unit of Infectious and Tropical Diseases, “Mater Domini” Teaching Hospital, Catanzaro, Italy.

**Keywords:** antimicrobial resistance, ESKAPE, Southern Italy, hospital units

## Abstract

This study evaluated the spread and possible changes in resistance patterns of ESKAPE bacteria to first-choice antibiotics from 2015 to 2019 at a third-level university hospital after persuasive stewardship measures were implemented. Isolates were divided into three groups (group 1, low drug-resistant; group 2, multidrug/extremely drug-resistant; and group 3, pan-resistant bacteria) and a *chi-squared* test (*χ*^2^) was applied to determine differences in their distributions. Among the 2,521 isolates, *Klebsiella pneumoniae* was the most frequently detected (31.1%). From 2015 to 2019, the frequency of isolates in groups 2 *and* 3 decreased from 70.1% to 48.6% (*χ*^2^ = 63.439; *p* < 0.0001). Stratifying isolates by bacterial species, for *K. pneumoniae*, the frequency of PDR isolates decreased from 20% to 1.3% (*χ*^2^ = 15.885; *p* = 0.003). For *Acinetobacter baumannii*, a statistically significant decrease was found in groups 2 *and* 3: from 100% to 83.3% (*χ*^2^ = 27.721; *p* < 0.001). Also, for *Pseudomonas aeruginosa* and *Enterobacter* spp., the frequency of groups 2 *and* 3 decreased from 100% to 28.3% (*χ*^2^ = 225.287; *p* < 0.001) and from 75% to 48.7% (*χ*^2^ = 15.408; *p* = 0.003), respectively. These results indicate that a program consisting of persuasive stewardship measures, which were rolled out during the time frame of our study, may be useful to control drug-resistant bacteria in a hospital setting.

## Introduction

Antimicrobial resistance (AMR) is one of the main threats to public health.^1^ It has been estimated that more than 670,000 infections occur every year and ∼33,000 people die due to bacteria resistant to antibiotics in Europe, with one-third of them in Italy.^1^ Indeed, the Antibiotic Resistance-Istituto Superiore di Sanità project found that Italy was severely affected by this problem.^2^ Particularly, bacteria belonging to the ESKAPE group (*i.e., Enterococcus faecium*, *Staphylococcus aureus*, *Klebsiella pneumoniae*, *Acinetobacter baumannii*, *Pseudomonas aeruginosa*, and *Enterobacter* spp.) represent a frequent cause of nosocomial infection,^3^ with increasing prevalence of multidrug resistance (MDR) to antibiotics, thereby reducing treatment options and increasing death rates because of treatment failure.^4^

Patient outcomes could be improved and spreading of MDR strains could be contained only with active monitoring of AMR and effective programs for antimicrobial stewardship, coupled with infection control.^5,6^ However, in the past years, insufficient attention had been paid to the problem, especially in our country.^7–9^ Therefore, the Ministry of Health set objectives and methods to control this problem,^10^ and several national programs have focused on measuring the size of the problem and promoting local actions.^11^ At regional levels, a process of finalizing guidelines has been initiated to increase the appropriateness of antimicrobial therapy.^12^

These guidelines should be interpreted as a deliverable of a process already ongoing, the effect of which could have been measured even before their publication. Accordingly, this study aimed to analyze data about epidemiology of resistant bacteria at the “*Mater Domini*” teaching hospital of Catanzaro (Calabria, Southern Italy).^13^ We hypothesized that ongoing interventions could have modified the trend of the relative prevalence of MDR bacteria, with specific reference to the ESKAPE group.

## Materials and Methods

### Samples

This descriptive, retrospective, longitudinal study analyzed the initial isolates of ESKAPE bacteria from any kind of samples for each patient admitted at the “*Mater Domini*” teaching hospital of Catanzaro from January 1, 2015, to December 31, 2019. According to the Italian legislation (GU Serie Generale no. 76 31/3/2008), due to the retrospective nature of the study and considering the absence of any demographic and clinical data of the patients, only a notification was due to the Ethical Committee which was sent on March 22, 2019. Samples were collected from urine, blood, wound, respiratory fluid (sputum and bronchoalveolar aspiration fluid), and other specimens; nasal and rectal swabs were excluded from the analysis aiming at reducing the effect of possible colonizations to increase the clinical relevance of the work. Samples were collected from patients admitted to four types of hospital units: medical units, surgical units, cardiac intensive care unit (CICU), and ICU. Pure bacterial cultures and antibiotic susceptibility testing were performed using an automated VITEK^®^ system (BioMérieux), although it is not considered the gold standard for some drugs.^14^

Susceptibility to antibiotics was evaluated based on the breakpoints of the European Committee on Antimicrobial Susceptibility Testing or EUCAST,^15^ and the intermediate level of sensitivity to antibiotics was considered resistant according to the European Centre for Disease Prevention and Control (ECDC) definitions.^16^

### Setting

The study was conducted at the “*Mater Domini*” teaching hospital, one of the two main hospitals in Catanzaro Province in the Calabria Region, Southern Italy. This is a third-level hospital in which critical patients from all regions are hospitalized. The number of beds and hospital admissions were 127 and 6,745 in 2015, respectively, and increased modestly over the calendar years (Table 1).

**Table 1.** Number of Beds and Hospital Admission at the “Mater Domini” Teaching Hospital During the Study Period

Hospital units	Year				
	2015	2016	2017	2018	2019
Medical Units					
Number of beds	59	59	87	87	85
Number of hospital admissions	3,596	3,611	4,398	4,581	4,475
Surgical Units					
Number of beds	54	54	78	78	78
Number of hospital admissions	2,452	2,095	2,528	2,843	2,890
Cardiac Intensive Care Unit					
Number of beds	6	6	12	12	12
Number of hospital admissions	226	183	83	78	78
Intensive Care Unit					
Number of beds	8	8	8	8	8
Number of hospital admissions	471	355	507	502	512
Total					
Number of beds	127	127	185	185	183
Number of hospital admissions	6,745	6,244	7,516	8,004	7,955

**FIG. 1.**
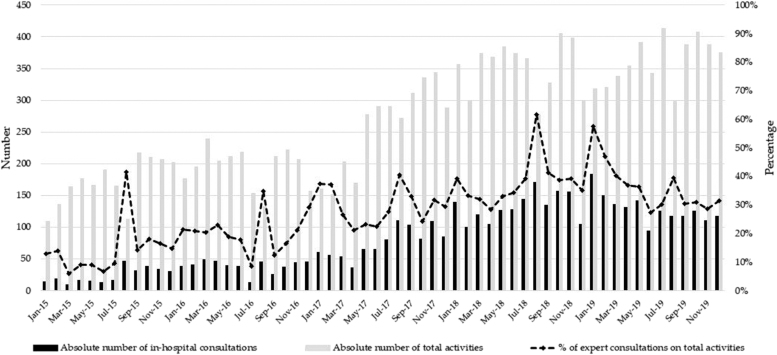
Activities of the Infectious Diseases unit during the study period. Total activities should be interpreted as the total number of days of in-hospital admissions, days of hospital admissions, outpatient consultations, and human immunodeficiency virus testing (including pretest and post-test counseling).

The number of activities of the units increases over time as in-hospital consultations for antimicrobial therapy become one of the most important parts of the workload (Fig. 1). Of note, patients were proactively evaluated after 48–72 hrs after the initial consultation to adjust antimicrobial therapy if necessary. Interventions to reduce the duration and increase the effectiveness of antibiotic treatment were put in place through evaluation of the clinical course, levels of procalcitonin, and microbiological results. During the study period, we also conducted several educational interventions and intensified infection control measures^17^ in line with national and regional guidelines.^10,12^ Accordingly, important educational workshops^18,19^ and projects^11,20,21^ have been conducted. Important topics as strategies to avoid/control outbreaks, the importance of hand hygiene, and practices for the prevention of surgical site infections were treated.

These events and projects were conducted not only by external expert in the field but also by nurses or medical doctors working in the hospital. Moreover, several nurses, especially those involved in the management of patients admitted to high infectious risk wards (*i.e.,* intensive care or infectious disease unit), attended a master diploma on management of infectious risk, which was started at “*Magna Graecia*” University since 2018.

### Definition of resistance

Bacterial isolates were classified according to the resistance profiles as indicated by the joined expert panel of ECDC and the U.S. Centers for Disease Control and Prevention.^16^ Particularly, group 1 (low resistant bacteria) comprised those without resistance to any class or with resistance to one molecule in ≤2 classes; group 2 (highly resistant bacteria) included MDR bacteria with resistance to ≥1 molecule in ≥3 different classes and extensively drug-resistant (XDR) bacteria with resistance to ≥1 molecule in all, but 2 or fewer classes; and group 3 (pandrug-resistant bacteria, PDR) consisted of those with resistance to all drugs and classes of antibiotics.

Merging MDR and XDR in the same category was applied either to increase statistical significance of the comparison due to the small number of isolates or to provide estimates for the worst-case scenario related to the presence of PDR bacteria. In fact, both MDR and XDR could be more easily treated than PDR bacteria, especially if one considers the availability of new drugs. Also, this classification was applied in our previous work^13^; however, to avoid any bias, a separate descriptive analysis was conducted to consider the relative prevalence of MDR and XDR bacteria as separate categories.

### Assessment parameters

According to guidelines,^16^ bacterial isolates were stratified according to the following parameters: year, type of hospital unit, organs, and systems. This study evaluated (i) number of isolates per bacterial species; (ii) frequency of bacteria and their distribution in groups (1, 2, or 3); (iii) frequency of Gram-negative isolates (*K. pneumoniae*, *A. baumannii*, *P. aeruginosa*, and *Enterobacter* spp.) in group 2 or 3, which were resistant to the following first-choice antibiotics: cephalosporins (*i.e.,* ceftazidime), carbapenems, colistin, amikacin, gentamicin, tigecycline, and piperacillin/tazobactam; (iv) frequency of antimicrobial drug resistance in group 2 Gram-positive isolates (*S. aureus* and *E. faecium*) to oxacillin, vancomycin, daptomycin, linezolid, and tigecycline for *S. aureus*, as well as to vancomycin, linezolid, and tigecycline for *E. faecium*; number and frequency of bacterial isolates in group 1, 2, or 3 by year (v), hospital units (vi), or organs and systems (vii); (viii) Gram-negative isolates with resistance to first-choice antibiotics by calendar years.

The resistance of *P. aeruginosa* to ertapenem or tigecycline was not considered in the analysis even if tested by the automated VITEK system (BioMérieux) method as it was due to intrinsic resistance to these drugs.

### Statistical analyses

Statistical analysis for qualitative data was performed using the chi-squared (*χ*^2^) test, and significance was set at *p* ≤ 0.05. Statistical analysis was performed between group 1 and groups 2 *and* 3 bacterial strains. Isolates were also analyzed by hospital units, as well as by organs and systems. To assess the trend of resistance patterns (group 1 and groups 2 *and* 3) during the five years analyzed, the *χ*^2^ test for trend (Cochrane-Armitage trend test) was applied. The *χ*^2^ test was considered not completely reliable (expected frequency below 1 or expected frequency less than 5 in more than 20% of cells as the condition for relying on the Gaussian approximation) in 44% of cases. Contingency tables of rows × columns from 2 × 3 to 2 × 7 were used.

## Results

### Bacterial species and patterns of resistance to antimicrobials

During the five study years, 2,521 bacterial isolates (ESKAPE species) were obtained. *K. pneumoniae* was the most represented species (31.1%), followed by *P. aeruginosa* (19.8%), *S. aureus* (18.6%), *Enterobacter* spp*. (*13.4%), *A. baumannii* (13.2%), and *E. faecium* (3.8%).

**FIG. 2.**
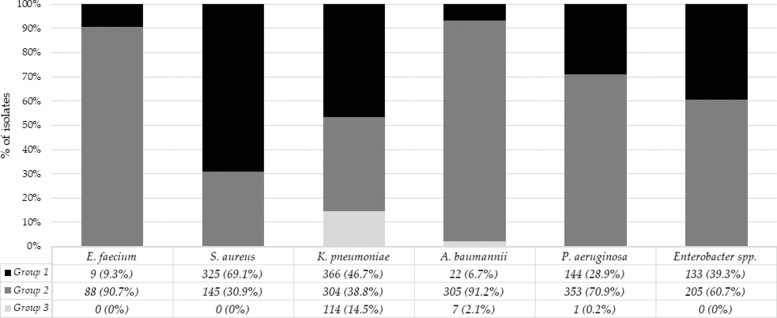
Overall number and frequency of bacterial isolates. Bacterial isolates are divided into three groups by antibiotic resistance patterns: group 1 (low resistant bacteria), group 2 (highly resistant bacteria), and group 3 (pan-resistant bacteria, PDR).

Distributions of bacterial species based on calendar years, hospital units, and types of samples are shown in Table 2. The frequency of bacterial isolates in group 1 or groups 2 *and* 3 differed significantly by species: *χ*^2^ = 401.179; *p* < 0.0001 (Fig. 2). Groups 2 *and* 3 bacteria were more common in Gram-negative bacteria (66.0%) than in Gram positive (41.1%) (*χ*^2^ = 113.653; *p* < 0.0001).

**Table 2.** Distribution of Bacterial Isolates by Years, Hospital Units, and Samples

	Bacterial species												Total	
	Enterococcus faecium		Staphylococcus aureus		Klebsiella pneumoniae		Acinetobacter baumannii		Pseudomonas aeruginosa		Enterobacter spp.			
Category	N	%	N	%	N	%	N	%	N	%	N	%	N	%
Year														
2015	10	10.3	82	17.4	145	18.5	43	12.9	74	14.9	44	13.0	398	15.8
2016	16	16.5	57	12.1	86	11.0	56	16.8	93	18.7	58	17.1	366	14.5
2017	26	26.8	108	23.0	184	23.5	48	14.4	94	18.9	69	20.5	529	21.0
2018	22	22.7	96	20.4	210	26.7	79	23.6	78	15.6	87	25.7	572	22.7
2019	23	23.7	127	27.1	159	20.3	108	32.3	159	31.9	80	23.7	656	26.0
TOT	97	100	470	100	784	100	334	100	498	100	338	100	2,521	100
Hospital Units														
Medical units	51	52.6	335	71.3	341	43.5	76	22.7	256	51.4	157	46.4	1,216	48.2
Surgical units	23	23.7	58	12.3	151	19.3	51	15.3	87	17.5	49	14.5	419	16.6
CICU	0	0	13	2.8	27	3.4	6	1.8	21	4.2	15	4.4	82	3.3
ICU	23	23.7	64	13.6	265	33.8	201	60.2	134	26.9	117	34.7	804	31.9
TOT	97	100	470	100	784	100	334	100	498	100	338	100	2,521	100
Sample														
Urine	41	42.4	22	4.7	229	29.2	29	8.7	84	16.9	51	15.1	456	18.1
Blood	14	14.4	84	17.9	94	12.0	37	11.1	25	5.0	24	7.1	278	11.0
Intravascular device	1	1	2	0.4	14	1.8	4	1.2	7	1.4	4	1.2	32	1.3
Wound swab	16	16.5	239	50.9	291	37.1	142	42.5	234	47.0	180	53.3	1,102	43.7
Respiratory sample	8	8.2	57	12.1	107	13.6	97	29.0	121	24.3	58	17.1	448	17.8
Other sample	17	17.5	66	14.0	49	6.3	25	7.5	27	5.4	21	6.2	205	8.1
TOT	97	100	470	100	784	100	334	100	498	100	338	100	2,521	100

**FIG. 3.**
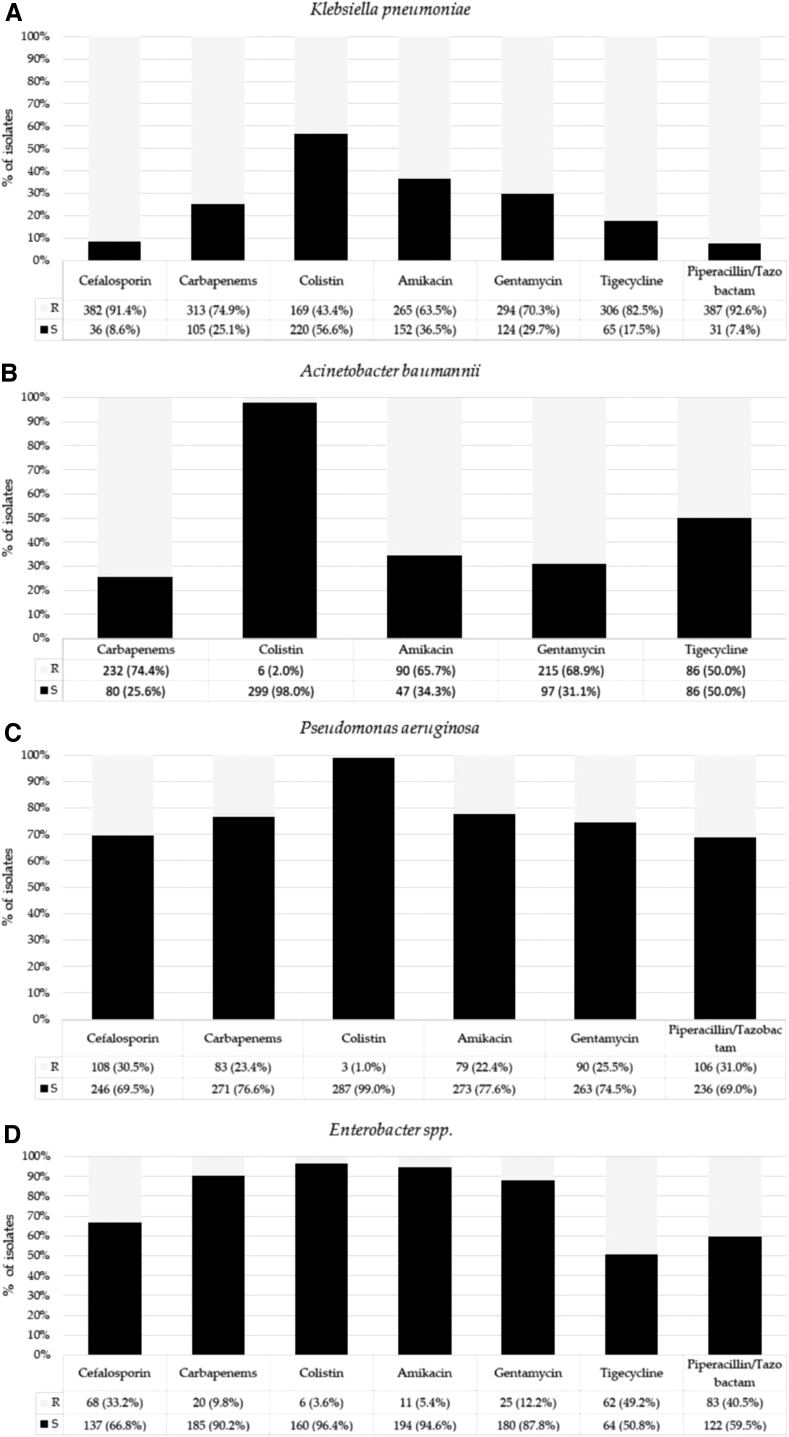
Overall frequency of antimicrobial drug resistance among MDR, XDR, and PDR isolates (Gram-negative bacteria). **(A–D)** show susceptibility rates to first-choice antibiotics among *Klebsiella pneumoniae*, *Acinetobacter baumannii*, *Pseudomonas aeruginosa*, and Enterobacter *spp*. isolates, respectively. MDR, multidrug resistance; XDR, extensively drug resistant; PDR, pandrug resistant.

Among the Gram-negative species in groups 2 *and* 3, the highest frequency of resistance to carbapenems was observed for *K. pneumoniae* (74.9%) and *A. baumannii* (74.4%), and the highest frequency of resistance to colistin was found for *K. pneumoniae* (43.4%) (Fig. 3). Among the Gram-positive species, no isolate was found in group 3. The highest frequency of isolates in group 2 was found for *E. faecium* (91%) (Fig. 2). Resistance to vancomycin was found in 12.5% of *E. faecium* isolates*,* and all strains were sensitive to linezolid and tigecycline. Resistance to oxacillin was found in 23% of *S. aureus*.

### Resistance patterns by calendar years

**FIG. 4.**
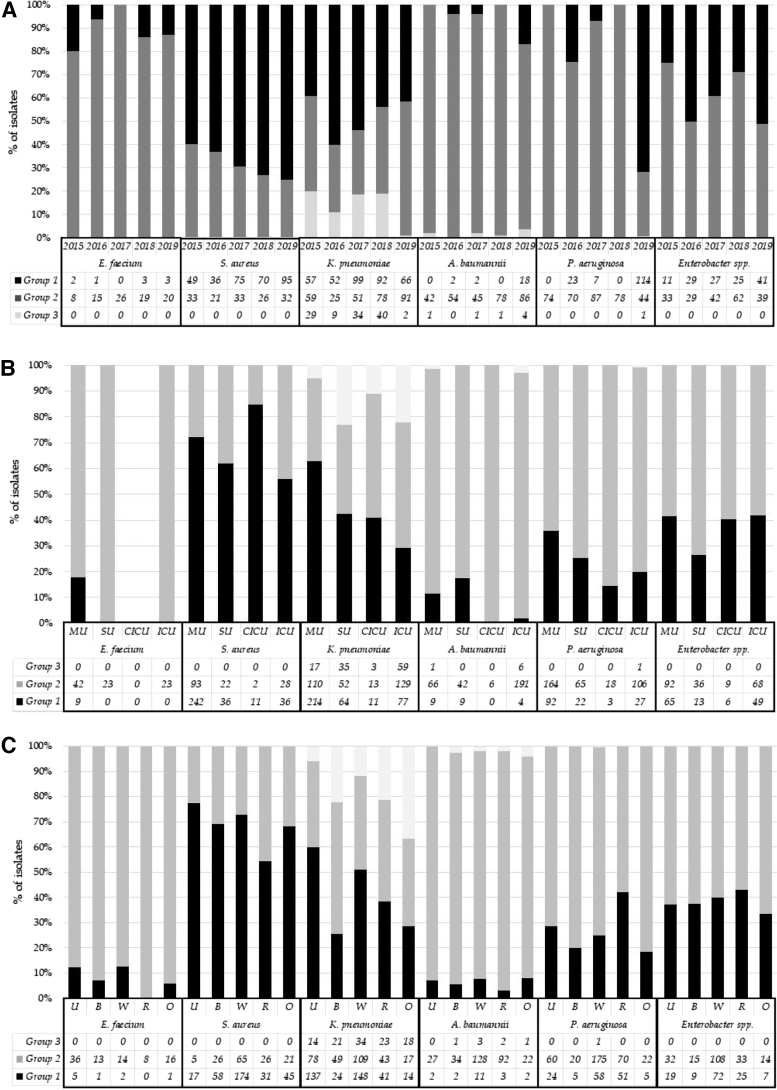
Number and frequency of bacterial isolates per calendar years **(A)**, hospital units **(B)**, and different sites **(C)**. Bacterial isolates are divided into three groups by antibiotic resistance patterns: group 1 (low resistant bacteria), group 2 (highly resistant bacteria), and group 3 (pan-resistant bacteria, PDR). MU, medical units; SU, surgical units; CICU, cardiac intensive care unit; ICU, intensive care unit; U, urine; B, blood; W, wound swabs; R, respiratory samples; O, other samples (miscellaneous).

Figure 4A shows the numbers and percentages of bacterial isolates in groups 1–3 based on calendar year. From 2015 to 2019, a significant change in the frequency of isolates was observed in both group 1 and groups 2 *and* 3 (*χ*^2^ = 63.439; *p* < 0.0001). While the frequency of isolates in group 1 increased, the frequency of isolates in groups 2 *and* 3 decreased from 70.1% in 2015 to 48.6% in 2019. Overall, the prevalence of Gram-negative isolates in groups 2 *and* 3 decreased during the study period. In the analysis of bacterial species, a significant reduction in PDR *K. pneumoniae* was observed *(χ*^2^ = 15.885; *p* = 0.003). As for *A. baumannii*, despite an overall increase in the number of isolates, a significant decrease in its frequency in groups 2 *and* 3 was observed (*χ*^2^ = 27.721; *p* < 0.001).

For *P. aeruginosa*, a reduction in the frequency in group 2 was observed (*χ*^2^ = 225.287; *p* < 0.001). For *Enterobacter* spp*.,* no isolate was included in group 3, and a reduction in its frequency in group 2 was observed (*χ*^2^ = 15.408; *p* = 0.003). A linear reduction trend was observed for *S. aureus* in group 2, from 40.2% in 2015 to 25.2% in 2019; however, the difference was not statistically significant (*χ*^2^ = 6.896; *p* = 0.141). Moreover, regarding the proportion of methicillin-resistant *S. aureus* (MRSA), we did not find any statistically significant trend from 2015 to 2019 (*χ*^2^ = 3.813; *p* = 0.431). For *E. faecium*, the trend was not statistically significant, and the frequency of isolates in groups was stable (*χ*^2^ = 5.082; *p* = 0.278).

In the analysis in which XDR and MDR were considered separate classes, for Gram-positive species, no isolate was detected in the XDR group. By contrast, for Gram-negative bacteria, the frequency of XDR over MDR, including XDR (group 2), over calendar years was as follows: (i) *K. pneumonia*, 23/59 (39%) in 2015, 7/25 (28%) in 2016, 5/51 (9.8%) in 2017, 27/78 (34.6%) in 2018, and 44/91 (48.4%) in 2019; (ii) *A. baumannii*, 25/42 (59.5%) in 2015, 36/54 (66.7%) in 2016, 23/45 (51.1%) in 2017, 60/78 (76.9%) in 2018, and 81/86 (94.2%) in 2019; (iii) *P. aeruginosa*, 15/74 (20.3%) in 2015, 22/70 (31.4%) in 2016, 9/87 (10.3%) in 2017, 9/78 (11.5%) in 2018, and 19/44 (43.2%) in 2019; (iv) *Enterobacter* spp., 4/33 (12.1%) in 2015, 2/29 (6.9%) in 2016, 1/42 (2.4%) in 2017, 8/62 (12.9%) in 2018, and 2/39 (5.1%) in 2019.

### Resistance patterns by hospital units

Figure 4B shows the number and percentage of bacterial isolates in groups 1–3 based on hospital units. The frequency of isolates in group 1 and groups 2 *and* 3 differed among hospital units (*χ*^2^ = 120.422; *p* < 0.0001). Particularly, the frequency of bacterial isolates in groups 2 *and* 3 was higher in the ICU (76%) than in surgical (65.6%) and medical (48.1%) units. Analysis of the distribution of Gram-negative and Gram-positive bacteria showed that for both frequencies, groups 2 *and* 3 were higher in the ICU than in other hospital units (Gram-positive bacteria: 56.6%, *χ*^2^ = 13.042; and Gram-negative bacteria: 78.1%, *χ*^2^ = 74.301; *p* < 0.001).

During the study period, the frequency of isolates in group 1 and groups 2 *and* 3 differed in ICU (*χ*^2^ = 15.236, *p* = 0.004), and a biphasic trend was observed with a lower resistance rate in 2019 (69.6%) than in 2015 (81.6%).

### Resistance patterns by sites of bacterial isolation (organ and system)

Figure 4C shows the number and percentage of bacterial isolates in groups 1–3 at different sites. The frequency of isolates with resistance to at least one molecule in groups 1*–*3 differed among organs and systems (*χ*^2^ = 19.943; *p* < 0.001). Particularly, the percentages of bacterial isolates in groups 2 *and* 3 were higher in respiratory (66.3%) and blood (66.1%) samples than in other samples (63.9%), wound swabs (57.8%), and urine (55.3%). Analysis of the distribution of Gram-negative and Gram-positive bacteria stratified by organs and systems showed that the frequency of group 2 was higher in urine than in the remaining samples for Gram-positive bacteria (65.1%; *χ*^2^ = 29.550; *p* < 0.001), while the frequency of groups 2 *and 3* was higher in the blood for Gram-negative bacteria (78.0%; *χ*^2^ = 47.766; *p* < 0.001).

### AMR to first-choice antibiotics for Gram-positive and Gram-negative isolates

#### Gram-positive isolates

Among Gram-positive isolates, very low levels of resistance to vancomycin, daptomycin, tigecycline, and linezolid were observed, and no statistically significant difference was found during the different study years. All isolates analyzed showed no resistance to linezolid, while 2.7% of isolates showed resistance to vancomycin (11/15 isolates were *E. faecium*). Notably, only 0.4% and 0.9% of isolates showed resistance to tigecycline and daptomycin, respectively.

#### Gram-negative isolates

**FIG. 5.**
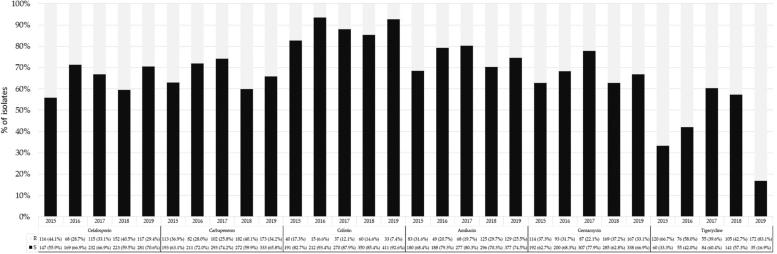
Number and frequency of Gram-negative bacterial isolates resistant to selected first-choice antibiotics by calendar years.

Figure 5 shows number and frequency of Gram-negative bacterial isolates resistant to first-choice antibiotics by calendar years. Among Gram-negative isolates (excluding *P. aeruginosa* considering its natural resistance), resistance to tigecycline decreased from 2015 (66.7%) to 2018 (42.7%), but a strong increase was observed in 2019 (83.1%) (*χ*^2^ = 102.371; *p* < 0.001). The frequency of resistance to cephalosporins (*χ*^2^ = 24.775), carbapenems (*χ*^2^ = 25.050), colistin (*χ*^2^ = 24.409), amikacin (*χ*^2^ = 17.897), and gentamicin (*χ*^2^ = 27.475) was significantly different during the study period (*p* < 0.001). For these molecules, a biphasic trend was observed during the study years, but the frequency of resistance was lower in 2019 than in 2015.

## Discussion

This study evaluated the trends in AMR from 2015 to 2019 in a large university hospital. It was very frequent to detect MDR strains, but it was rarer to detect strains resistant to all available antibiotics. Interestingly, most bacteria showed a decrease in AMR, while the effect was limited for some bacteria. In contrast, many recent studies have shown an increase in AMR, particularly for Gram-negative bacteria.^22,23^ Since this work aimed to further explore the possible impact of interventions on controlling the spread of MDR bacteria at our setting, as an update of a previous study,^13^ the continuing decrease over time in the relative prevalence of MDR bacteria suggests that greater attention to the AMR issue has a positive impact. Consistently, the antibiotic consumption at our institute was lower than that at other hospitals in the Calabria Region, especially for ceftriaxone, meropenem, and piperacillin/tazobactam,^12^ even if the effect of reduction in antimicrobial consumption on AMR has been demonstrated to be inconsistent for all drugs and limited in time.^24^

Moreover, future studies should compare AMR in relation to the use of antibiotics across different centers in our setting, since no information is available to support consistently higher MDR rates in hospitals with heavier consumptions of antibiotics. Our data indicate that AMR remains a significant problem, especially in the ICU. Indeed, despite an overall decrease in the relative prevalence of MDR bacteria, AMR in the ICU remains a challenge: over 50% of bacteria were detected in groups 2 *and* 3 with important consequences in therapy prescription. Moreover, as reported in previous studies,^13,17^ most of the isolated bacteria were Gram negative, were more often MDR than Gram-positive ones (66.0% vs. 41.1%), and often occurred in deep sites (blood/respiratory system).^25^

Among Gram-negative bacteria in the ESKAPE group, *K. pneumoniae* was the most represented species (40.1%), while *A. baumannii* was the species with the highest frequency of MDR isolates (93.4%), including XDR (group 2) and PDR (group 3) (Fig. 2). Indeed, *A. baumannii* represents a challenging clinical problem for the following reasons: (i) although 90% of the isolates are susceptible to colistin, its use is often burdened by kidney and neurotoxicity, and its pharmacokinetic profile is not optimal, especially for pneumonia treatment;^26^ and (ii) the number of active drugs is limited, ranging from 25.6% for carbapenems to 50% for tigecycline, a drug whose systemic bioavailability is suboptimal and treatment of pneumonia may require increasing dosages.^27^

Interestingly, however, even for *A. baumannii*, a lower frequency of resistance to carbapenems was observed in the years 2015–2019 (74.4%) compared to the years 2010–2014 in our previous study, in which isolates in groups 2 *and* 3 showed about 100% of resistance to meropenem;^13^ it has therefore become closer to the average estimate at the national level (about 80% of resistance reported by Istituto Superiore di Sanità).^2^ In addition, in *K. pneumoniae*, the highest frequency of PDR strains (group 3) was found (14.5%); these strains showed the highest frequency of resistance to colistin and tigecycline, which was even more than that in other institutions,^28,29^ and this could be correlated with an increase in admissions of patients from other clinical centers, particularly for colistin in the ICU, which became a reference center for care of patients with severe respiratory infections in our region.

Therefore, the availability of new drugs, such as ceftazidime/avibactam or cefiderocol, appears to be important for controlling these infections.^30,31^ Despite reduction of frequency of groups 2 *and* 3 of bacterial isolates over calendar years, the relative frequency of XDR in group 2 remained a problem, accounting for over 40% of isolates with MDR, especially for *K. pneumoniae*, *A. baumannii*, and *P. aeruginosa*, meaning that efforts should be pursued to use new drugs appropriately and support antimicrobial stewardship programs with sustained interventions.

*P. aeruginosa* and *Enterobacter* spp. were the least found among the Gram-negative isolates. Group 3 isolates were virtually absent, confirming the results of our previous analysis, at least for *P. aeruginosa*.^13^ Moreover, for *P. aeruginosa*, a decrease in the frequency of group 2 was observed (especially in 2019, in which only 28.3% isolates were MDR/XDR), and a parallel increase in group 1 *P. aeruginosa* was found, especially in 2019 (Fig. 4). However, the frequency observed was higher than that in other Italian and European datasets.^1^ Moreover, the reasons for dramatic increase of group 1 *P. aeruginosa* in 2019 compared to other bacteria are difficult to explain because it may either be an effect of the adopted measures of antimicrobial stewardship and infection control or this variation occurred by chance due to the low number of the bacterial isolates analyzed.

In any case, optimal management of *P. aeruginosa* infections, especially for MDR or XDR strains, is still controversial.^32^ Ceftolozane/tazobactam may be the best therapeutic option, and a high success rate of cure has also been reported in the case of off-label use.^33^ Moreover, regarding *Enterobacter* spp*.,* the following considerations may indicate that it is not an important problem compared with other Gram-negative ESKAPE species: (i) in the whole period, the burden of the problem appeared to be limited, accounting for only 13.4% of isolates; (ii) the frequency of group 1 isolates was higher compared with *P. aeruginosa* and *A. baumannii*; and (iii) PDR isolates were not observed. However, high frequency of resistance strains was observed for tigecycline (49.2%), piperacillin/tazobactam (40.5%), and cephalosporins (33.2%), thus limiting the use of carbapenem-sparing strategies.

During the study period, Gram-positive strains appeared to represent a smaller problem compared to Gram-negative strains with high levels of sensitivity to vancomycin, daptomycin, tigecycline, and linezolid. However, only a trend toward reduction of MRSA was found in this study (from 23.2% in 2015 to 19.7% in 2019), compared to 34% across Italy, which remained stable in the same period.^2^ In conclusion, even though the burden of Gram-positive organisms with AMR was less compared with Gram-negative bacteria in absolute terms (with favorable rates of MRSA over time compared to the rest of Italy), this problem should not be disregarded since the National Program for the Fight of AMR set a bigger reduction of >10% MRSA from 2016 to 2020 as the main target.^10^

This study has several limitations. First, the clinical validation of the official definition to rank AMR into groups in this study and previous studies^13,16^ is limited.^34^ With this definition, all antibiotics are weighted equally independent from their pharmacokinetic or toxicity profiles and without considering the individual impact of MDR/XDR strains on mortality.^35,36^ However, definitions of “difficult-to-treat” resistance (DTR), proposed by several authors,^34,37,38^ are not yet incorporated in official guidelines for epidemiological purposes, and clinical utility for prediction of mortality has not been uniformly validated.^39^ Moreover, considering the availability of new drugs, including cefiderocol, ceftazidime/avibactam, or ceftolozane/tazobactam, treatment of infections due to bacteria with MDR or DTR may become more effective, and the above definitions may require amendment.

Notably, our aim was mainly epidemiological rather than clinical in nature, for example, evaluating the appropriateness of antibiotic therapy or its impact on mortality. Second, this study was monocentric and the sample size is limited; however, our institution could serve as a sentinel site to determine the rates of AMR in a large part of the region where many patients came from. Third, phenotypic results were determined using the automated VITEK system (BioMérieux) because this is the standard method used in clinical practice, even though it is uncertain whether this is the best system to predict clinical response; for instance, the high rate of resistance of *K. pneumoniae* to colistin has not be confirmed with other methods, such as broth microdilution.^14^

Moreover, considerations of resistance phenotypes, inference, inferring, and interpretative reading suggest that the VITEK system, as a unique system, is still questionable^40–46^ and not uniformly used worldwide, for instance, in some Asian countries.^47^ Therefore, future studies should measure resistance using more appropriate methods. Fourth, molecular mechanisms or genes underlying resistance have not been investigated. The related researches may be useful.

## Conclusions

AMR remains a major public health problem. Our results support that antimicrobial stewardship activities over time may help prevent the development of resistance and should be further examined. A network with shared local and regional data is likely to be very useful, especially considering that patients are frequently transferred from one institution to another one, so the availability of information on previous MDR isolates has a high relevance both to guide the choice of therapy and institute infection control measures, especially in patients with severe infections due to bacteria characterized by MDR profiles.

## Data Availability

The data that support the findings of this study are available upon request from the corresponding author. The data are not publicly available because of privacy or ethical restrictions.
